# Lower Bounds on Anderson-Localised Eigenfunctions on a Strip

**DOI:** 10.1007/s00220-022-04346-5

**Published:** 2022-03-26

**Authors:** Ilya Goldsheid, Sasha Sodin

**Affiliations:** School of Mathematical Sciences, Queen Mary University of London, London, E1 4NS UK

## Abstract

It is known that the eigenfunctions of a random Schrödinger operator on a strip decay exponentially, and that the rate of decay is not slower than prescribed by the slowest Lyapunov exponent. A variery of heuristic arguments suggest that no eigenfunction can decay faster than at this rate. We make a step towards this conjecture (in the case when the distribution of the potential is regular enough) by showing that, for each eigenfunction, the rate of exponential decay along any subsequence is strictly slower than the fastest Lyapunov exponent, and that there exists a subsequence along which it is equal to the slowest Lyapunov exponent.

## Introduction

Let $W \ge 1$, and let *V*(*n*), $n \ge 0$, be independent, identically distributed random variables taking values in the space of $W \times W$ real symmetric matrices, so that

1 $$\begin{aligned} {\mathbb {E}} \Vert V(n)\Vert ^\eta < \infty \quad \text {for some }\eta > 0, \end{aligned}$$

and the support ${\mathcal {S}}$ of the distribution of *V*(*n*) is sufficiently rich, say, in the following sense:2The main example (the Schrödinger case) is

3 $$\begin{aligned} V(n)_{\alpha ,\alpha '} = {\left\{ \begin{array}{ll} 1, &{}|\alpha -\alpha '| = 1 \\ v_{n,\alpha }, &{}\alpha = \alpha ' \end{array}\right. } \qquad \text {(and 0 otherwise),}\end{aligned}$$

where $\{v_{n,\alpha }\}_{n \in {\mathbb {Z}}_+, \alpha \in \{1,\cdots ,q\}}$ are independent, identically distributed real-valued random variables not concentrated at one point and having ${\mathbb {E}} |v_{n,\alpha }|^\eta <\infty$.

We are interested in the spectral properties of the random operator *H* on $\ell _2({\mathbb {Z}}_+ \rightarrow {\mathbb {C}}^W)$, defined as follows:

$$\begin{aligned} (H \psi )(n) = {\left\{ \begin{array}{ll} \psi (n+1) + V(n) \psi (n) + \psi (n-1), &{} n \ge 1\\ \psi (1) + V(0) \psi (0), &{}n = 0. \end{array}\right. }\end{aligned}$$

On an event of full probability, *H* exhibits Anderson localisation which manifests itself in the following spectral properties: the spectrum of *H* is pure point, and the eigenfunctions decay exponentially, meaning that there exists a deterministic $\gamma > 0$ such that for each eigenfunction $\psi$ of *H*

4 $$\begin{aligned} \limsup _{n \rightarrow \infty } \frac{1}{n} \log \Vert \psi (n)\Vert \le - \gamma . \end{aligned}$$

where $\Vert \cdot \Vert$ denotes the Euclidean norm in ${\mathbb {R}}^W$.

For $W=1$, the pure point nature of the spectrum was first established in [18], and exponential decay – by Molchanov in [32]; see further Kunz and Souillard [26]. In these works, it was assumed that the distribution of the potential is absolutely continuous with bounded density, The case of singular potentials was settled by Carmona, Klein, and Martinelli [6]. For $W>1$ (Schrödinger case) with absolutely continuous distribution of the potential, the pure point nature of the spectrum was first proved in [15], and exponential decay – by Lacroix in [27, 28]. The general Schrödinger case was settled by Klein, Lacroix and Speis in [25], building on [17]. The argument of [25] can be extended to the general situation (2), once the result of [16] (discussed below) is taken into account; an alternative argument avoiding multi-scale analysis and applicable to the general model (1) (and also to its further generalisation allowing for random hopping) is given in [31]. In this paper, we do not discuss Anderson localisation in dimension $d > 1$, and refer to the works of Fröhlich and Spencer [9] and Aizenman and Molchanov [2] and also to the monograph of Aizenman and Warzel [3].

A more precise version of the relation (4) can be stated in terms of the Lyapunov exponents associated with *H*. For $\lambda \in {\mathbb {R}}$, define the one-step transfer matrices

$$\begin{aligned} T_n (\lambda ) = \left( \begin{array}{cc} \lambda - V(n) &{} - \mathbbm {1} \\ \mathbbm {1} &{} 0 \end{array} \right) \in {\text {Sp}}(2W, {\mathbb {R}}) \qquad (n \ge 0) \end{aligned}$$

and the multi-step transfer matrices

$$\begin{aligned} \Phi _{n, n'}(\lambda ) = T_{n-1}(\lambda ) \cdots T_{n'}(\lambda ), \quad \Phi _n(\lambda ) = \Phi _{n,0}(\lambda ) \qquad (n > n' \ge 0). \end{aligned}$$

The Lyapunov exponents $\gamma _1(\lambda ) \ge \gamma _2(\lambda ) \ge \cdots \ge \gamma _{2W}(\lambda )$ are defined as

$$\begin{aligned} \gamma _j(\lambda ) = \lim _{n \rightarrow \infty } \frac{1}{n} {\mathbb {E}} \log s_j(\Phi _n(\lambda )), \end{aligned}$$

where $s_j$ stands for the *j*-th singular value. According to a general result of Furstenberg and Kesten [10], one has

5 $$\begin{aligned} \forall \lambda \in {\mathbb {R}} \,\,\,\,\, {\mathbb {P}} \left\{ \gamma _j(\lambda ) = \lim _{n \rightarrow \infty } \frac{1}{n} \log s_j(\Phi _n(\lambda )) \right\} = 1.\end{aligned}$$

Due to the symplectic structure, $\gamma _{2W+1-j}(\lambda ) = - \gamma _j(\lambda )$ for $j =1, \cdots , W$.

Following precursory work by Tutubalin (see the survey [34]) and Virtser [37], Guivarc$'$h and Raugi showed [20] that if

6 $$\begin{aligned} \left[ \begin{array}{ll} &{}\hbox {the action of the semigroup generated by the support } {\mathcal {S}}_\lambda \hbox { of } T_n(\lambda ) \\ &{}\hbox {on } {\mathbb {R}}^{2W} \hbox { and its wedge powers is strongly irreducible and contractive,} \end{array} \right. \end{aligned}$$

then the Lyapunov exponents are distinct:

7 $$\begin{aligned} \gamma _1(\lambda )> \gamma _2(\lambda ) \cdots>\gamma _W(\lambda ) > 0. \end{aligned}$$

In the case (3) with absolutely continuous distribution of $v_{n,\alpha }$, the condition (6) was verified in [27], while in [15] (7) was directly established using the results of [34]. In [17], the following general theorem is proved: (6) (and consequently also (7)) holds if8It was also shown in [17] that in the Schrödinger case (3) one has (8) for any $\lambda \in {\mathbb {R}}$. In [16], a general method to compute the Zariski closure of the group generated by the support of $T_n(\lambda )$ was developed; one of its consequences is that (8) holds for any $\lambda \in {\mathbb {R}}$ also in the generality of (2).

Now we can state the full result of Klein, Lacroix and Speis [25] (in the current setting, covered by [31]): there is an event of full probability on which each eigenpair $H\psi = \lambda \psi$ satisfies

9 $$\begin{aligned} \limsup _{n \rightarrow \infty } \frac{1}{n} \log \Vert \psi (n)\Vert \le - \gamma _W(\lambda ). \end{aligned}$$

A variety of heuristic arguments indicate that (9) should be sharp in the following strong sense: there is an event of full probability on which each eigenpair $H\psi = \lambda \psi$ satisfies

10 $$\begin{aligned} \text {(conjecture)}\qquad \liminf _{n \rightarrow \infty } \frac{1}{n} \log (\Vert \psi (n)\Vert + \Vert \psi (n+1)\Vert ) \ge - \gamma _W(\lambda ), \end{aligned}$$

which, in conjuction with (9), implies the existence of a limit equal to $-\gamma _W(\lambda )$. For example, the Fermi Golden Rule leads one to believe that eigenfunctions violating (10) are unstable under perturbation. From the point of view of random matrix products, an eigenfunction decaying at a rate faster than $\gamma _W$ indicates a non-generic intersection between the *W*-dimensional space of initial conditions with the *W*-dimensional Oseledec subspace of decaying solutions in ${\mathbb {R}}^{2W}$.

The relation (10) was repeatedly conjectured at least since the 1980s; however, we are not aware of any rigorous results improving on the trivial bound

11 $$\begin{aligned} \liminf _{n \rightarrow \infty } \frac{1}{n} \log (\Vert \psi (n)\Vert + \Vert \psi (n+1)\Vert ) \ge - \gamma _1(\lambda ) \end{aligned}$$

(which follows from a general result of Craig and Simon [7], or from its quantitative version, stated as Lemma 2.2 below). The main difficulty comes from the fact that, although for each fixed energy $\lambda$ the probability to have an eigenfunction which decays at a rate faster than $\gamma _W(\lambda )$ is zero, one can not use the union bound over the uncountable set of all real $\lambda$.

In this paper we make a step towards (10) by improving upon (11) (in the case when the distribution of potential is regular enough). To state the results precisely, we introduce some notation. Let ${\mathcal {E}}(H) = \{ (\lambda , \psi ) \}$ be the collection of eigenpairs of *H*, with the normalisation $\Vert \psi (0)\Vert =1$ (the choice of the sign is not important for us, and spectral multiplicity is known to be a null event). For $\gamma > 0$ and a bounded interval $I \subset {\mathbb {R}}$, consider the two realisation-dependent sets:

12 $$\begin{aligned} {\text {Fast}}^+(\gamma ; I)= & {} \left\{ \lambda \in I: \exists (\lambda , \psi ) \in {\mathcal {E}}(H),\, \liminf _{n \rightarrow \infty } \frac{\log (\Vert \psi (n)\Vert + \Vert \psi (n+1)\Vert ) }{n} \le - \gamma \right\} , \nonumber \\ {\text {Fast}}^-(\gamma ; I)= & {} \left\{ \lambda \in I: \exists (\lambda , \psi ) \in {\mathcal {E}}(H),\, \limsup _{n \rightarrow \infty } \frac{\log (\Vert \psi (n)\Vert + \Vert \psi (n+1)\Vert ) }{n} \le - \gamma \right\} . \nonumber \\ \end{aligned}$$

These sets consist of the eigenvalues for which the corresponding eigenvector decays at rate $\ge \gamma$ (along a subsequence, or uniformly). We note that there is no simple way to define the sets as random variables on the underlying probability space (see Kendall [24] and Tsirelson [36] for possible frameworks to address such questions); this does not cause problems since we only work with the measureable events $\{ {\text {Fast}}^\pm (\gamma ; I) = \varnothing \}$ and $\{ {\text {Fast}}^\pm (\gamma ; I) \ne \varnothing \}$ (which in fact lie in the tail $\sigma$-algebra).

For $\lambda$ in the spectrum $\sigma (H)$ of *H*, define the deterministic quantities

13 $$\begin{aligned} \gamma _*^\pm (\lambda ) = \inf \Big \{ \gamma> 0 : \, \exists r > 0, \, {\mathbb {P}} \left\{ {\text {Fast}}^\pm (\gamma , (\lambda - r, \lambda + r)) \ne \varnothing \right\} = 0 \Big \} . \end{aligned}$$

Roughly speaking, the functions $\gamma _*^\pm (\lambda )$ measure the fastest possible decay of an eigenfunction in the vicinity of $\lambda$ (recall that $\gamma _j(\lambda )$ are continuous, cf. below, and that $\sigma (H)$ is almost surely equal to a deterministic set). In this notation, (9) and (11) imply that

14 $$\begin{aligned} \gamma _1(\lambda ) \ge \gamma _*^{+}(\lambda ) \ge \gamma _*^{-}(\lambda ) \ge \gamma _W(\lambda ),\end{aligned}$$

whereas the conjecture (10) stipulates that the last two inequalities are in fact equalities: $\gamma _*^\pm \overset{\text {\tiny conj}}{=} \gamma _ W$. The results below show that the first inequality in (14) is strict (for $W \ge 2$), whereas the last one is an equality, at least, if one asumes

### Assumption 1.1

(a) The distribution of *V*(*n*) is compactly supported on a real-analytic submanifold ${\mathcal {M}}$ in the space of symmetric $W \times W$ matrices, and is absolutely continuous with bounded density with respect to the $(\dim {\mathcal {M}})$-dimensional Lebesgue measure on ${\mathcal {M}}$; (b) for each $\lambda \in {\mathbb {R}}$ the image of ${\mathcal {M}}$ under

$$\begin{aligned} V \mapsto \left( \begin{array}{cc} \lambda - V &{} - \mathbbm {1} \\ \mathbbm {1} &{} 0 \end{array} \right) \end{aligned}$$

generates ${\text {Sp}}(2W, {\mathbb {R}})$ as a Lie group.

### Remark 1.2

Assumption 1.1 implies both (1) and (8).

### Remark 1.3

In the Schrödinger case (3), Assumption 1.1 is satisfied if the random variables $v_{n,\alpha }$ are bounded and their distribution is absolutely contiunous with bounded density (see [29, Section 1.4]).

### Theorem 1

Let $W \ge 3$. If Assumption 1.1 holds, then $\gamma _*^{+}(\lambda ) \le \gamma _{*,1}(\lambda )$ for $\lambda \in \sigma (H)$, where $\gamma _{*,1}(\lambda )$ is the unique solution of the equation

15 $$\begin{aligned} \big ( (W-1) \gamma - \sum _{j=1}^{W-1}\gamma _j(\lambda )\big )_+ + \gamma = \gamma _1(\lambda ). \end{aligned}$$

Here $x_+ = \max (x, 0)$. We observe that for $W \ge 3$ $\gamma _{*,1}(\lambda ) < \gamma _1(\lambda )$, hence (15) indeed improves on (11). For $W = 2$, $\gamma _{*,1} = \gamma _1(\lambda )$; however, we prove

### Theorem 2

Let $W = 2$. If Assumption 1.1 holds, then $\gamma _*^{+}(\lambda ) \le \frac{2 \gamma _{1}(\lambda ) + \gamma _2(\lambda )}{3}$ for all $\lambda \in \sigma (H)$.

As for $\gamma _*^{-}$, our methods yield the optimal result:

### Theorem 3

Let $W \ge 2$. If Assumption 1.1 holds, then $\gamma _*^{-}(\lambda ) = \gamma _{W}(\lambda )$ for all $\lambda \in \sigma (H)$.

The following corollary summarises our main conclusions:

### Corollary 1.4

Let $W \ge 2$. If Assumption 1.1 holds, then

16 $$\begin{aligned} \gamma _W(\lambda ) = \gamma _*^{-}(\lambda ) \le \gamma _*^{+}(\lambda ) < \gamma _{1}(\lambda ) \end{aligned}$$

for all $$\lambda \in \sigma (H)$$.

In the proofs, we repeatedly use the following argument, inspired by the work of Kakutani [23] and its ramifications by Spencer and Aizenman [1], to estimate the probability of exceptional events. Suppose we want to bound ${\mathbb {P}}\left\{ A \ne \varnothing \right\}$, where *A* is a random subset of, say, the interval [0, 1]. Suppose we find $\eta \in (0, 1]$ and a random superset $A^{+} \supset A$ with the following properties: (a) for each $\lambda \in [0, 1]$, ${\mathbb {P}} \left\{ \lambda \in A^{+}\right\} \le p$ (“single-energy bound”); (b) if $\lambda \in A$ and $|\lambda ' - \lambda | < \eta$, then $\lambda ' \in A^{+}$ (“propagation estimate”). Then the Chebyshev inequality and the Fubini theorem yield:

$$\begin{aligned} {\mathbb {P}} \left\{ A \ne \varnothing \right\} \le {\mathbb {P}} \left\{ {\text {mes}} (A^+ \cap [0,1]) \ge \eta \right\} \le \frac{1}{\eta }{\mathbb {E}} {\text {mes}} (A^+ \cap [0,1]) \le \frac{p}{\eta }.\end{aligned}$$

The paper is organised as follows. Some preliminary estimates are collected in Sect. 2. In Sects. 3 and 4 we prove Theorems 2 and 3, respectively. In Sect. 5 we discuss the prospects of improving the bounds in Theorems 1 and 2, and point out the connection to the problem, going back to [13, 14] and recently studied by Gorodetski and Kleptsyn [19], of uniform convergence to the Lyapunov exponents, i.e. whether the quantifier $\forall \lambda$ in (5) can be inserted inside the curly brackets. We also prove Proposition 5.1, which is an ${\text {Sp}}(2W, {\mathbb {R}})$-counterpart of one of the results of [19]. The proof of Theorem 3 in Sect. 6 makes use of this proposition.

We conclude this introduction with two remarks. First, we have chosen to present the arguments for the one-sided strip ${\mathbb {Z}}_+ \times \{1, \cdots , W\}$; similar arguments can be applied to the two-sided strip ${\mathbb {Z}} \times \{1, \cdots , W\}$. Second, it is possible that Assumption 1.1 can be somewhat relaxed, and that a refinement of the current methods could be applicable when the invariant measure (describing the limiting distribution of the unitary matrices in the singular value decomposition of the transfer matrices $\Phi _n$) is absolutely continuous with bounded density with respect to the Haar measure on the compact symplectic group, or at least enjoys the Frostman property (upper bound on the measure of every ball by a power of the radius) with a sufficiently large exponent. On the other hand, it is known (see [21] for the case $W=1$) that for singular distributions of *V*(*n*) the invariant measure may be supported on lower-dimensional subsets of the symplectic group. Extending our results to such cases would require additional ideas.

## Preliminaries

**Convergence to the Lyapunov exponent** Assume that (6) holds at some $\lambda$. Then (6) also holds in a neighbourhood of $\lambda$, and then (see e.g. [25, Corollary 2.5]) the Lyapunov exponents $\gamma _j(\lambda )$ are continuous at $\lambda$. For each $\epsilon > 0$, let $r_\epsilon (\lambda )\in (0, 1/2]$ be such that

17 $$\begin{aligned} \forall \lambda ' \in (\lambda - r_\epsilon (\lambda ), \lambda + r_\epsilon (\lambda )) \,\, \forall 1 \le j \le W \,\,\,\, |\gamma _j(\lambda ') - \gamma _j(\lambda )| < \epsilon . \end{aligned}$$

The following large deviation estimate goes back to the work of Le Page [30].

### Lemma 2.1

(see [8, 5, Section V.6]) Assume (1). Let $I \subset {\mathbb {R}}$ be a finite interval such that (6) holds for all $\lambda \in I$. Then there exist $C>0$ and $c>0$ such that for each $\lambda \in I$, $1 \le j \le W$, $\epsilon \in (0, 1]$, and $n \ge 1$

18 $$\begin{aligned} {\mathbb {P}}\left\{ \left| \frac{1}{n} \log s_j(\Phi _n(\lambda )) - \gamma _j(\lambda ) \right| \ge \epsilon \right\} \le C \exp (-c \epsilon ^2 n).\end{aligned}$$

The arguments leading to the following corollary of Lemma 2.1 are also well known (for $W = 1$, see e.g. Jitomirskaya and Zhu [22, Section 5]; we also mention a result of Craig–Simon [7, Theorem 2.3], which is not quantitative, but on the other hand holds in more general setting).

### Lemma 2.2

Assume (1). Suppose $\lambda \in {\mathbb {R}}$ is such that (6) holds. Then there exist $C>0$ and $c>0$ such that for each $1 \le j \le W$, $\epsilon \in (0, 1]$, and $n \ge 1$

$$\begin{aligned}&{\mathbb {P}}\left\{ \exists \lambda ' \in (\lambda - r_\epsilon (\lambda ), \lambda + r_\epsilon (\lambda )) \,\, : \,\, \frac{1}{n} \sum _{i=1}^j\right. \\&\quad \left. \log s_i(\Phi _n(\lambda ')) \ge \sum _{i=1}^j \gamma _i(\lambda ) + 2j \epsilon \right\} \le C n \exp (-c \epsilon ^2 n).\end{aligned}$$

### Proof

If $n \le 100 W^2$ or $\epsilon ^2 \le 100 \log n / n$, we can ensure the desired inequality by adjusting the constants, therefore we assume that $n > 100 W^2$ and $\epsilon ^2 \ge 100 \log n / n$. Consider the *j*-th exterior power $\Phi _n(\lambda ')^{\wedge j}$ of $\Phi _n(\lambda ')$, so that

$$\begin{aligned}\log \Vert \Phi _n(\lambda ')^{\wedge j} \Vert = \sum _{i=1}^j \log s_j(\Phi _n(\lambda ')).\end{aligned}$$

Each matrix element $p(\lambda ')$ of $\Phi _n(\lambda ')^{\wedge j}$ (where *p* runs in a finite set *P* enumerating the matrix elements) is a polynomial of degree $\le j n \le W n$ in $\lambda$. Now we use the following result of Bernstein [4], although we require much less than its full strength (in place of the logarithmic dependence on the degree with a precise constant, we could do with any prefactor growing slower than exponentially): for any polynomial *q* of degree *n*

$$\begin{aligned} \max _{|\lambda | \le 1} |q(\lambda )| \le C_n \max _{\alpha \in \{0,1,\cdots ,n\}} |q(\cos (\pi \frac{\alpha +\frac{1}{2}}{n+1}))|, \quad \text {where} \quad C_n = (1+ o(1)) \, \frac{2}{\pi }\log n. \end{aligned}$$

Returning to our setting, let

$$\begin{aligned} \lambda _\alpha = \lambda + r_\epsilon (\lambda ) \cos (\pi \frac{\alpha +\frac{1}{2}}{Wn+1}), \quad 0 \le \alpha \le Wn~;\end{aligned}$$

then we have for any $p \in P$:

19 $$\begin{aligned} \max _{\lambda ' \in (\lambda - r_\epsilon (\lambda ), \lambda + r_\epsilon (\lambda ))} |p(\lambda )| \le C \log (Wn) \max _{\alpha \in \{0,1,\cdots ,Wn\}} |p(\lambda _\alpha )| \le e^{\frac{\epsilon n}{3}} \max _{0 \le \alpha \le Wn} |p(\lambda _\alpha )|.\nonumber \\ \end{aligned}$$

By Lemma 2.1 and the choice of $r_\epsilon$,

$$\begin{aligned} {\mathbb {P}} \left\{ |p(\lambda _\alpha )| \ge \exp \left\{ n \left[ \sum _{i=1}^j \gamma _i(\lambda ) + \frac{4j}{3} \epsilon \right] \right\} \right\} \le C' \exp (-c' \epsilon ^2 n).\end{aligned}$$

Thus by (19)

$$\begin{aligned}&{\mathbb {P}} \left\{ \max _{p \in P}\max _{\lambda ' \in (\lambda - r_\epsilon (\lambda ), \lambda + r_\epsilon (\lambda ))} |p(\lambda ')| \ge \exp \left\{ n \left[ \sum _{i=1}^j \gamma _i(\lambda ) + \frac{5j}{3} \epsilon \right] \right\} \right\} \\&\quad \le C'' n \exp (-c' \epsilon ^2 n). \end{aligned}$$

Finally, $\Vert \Phi _n(\lambda ')\Vert \le C \max _{p \in P} |p(\lambda ')| \le e^{\epsilon n /3} \max _{p} |p(\lambda ')|$, and this completes the proof. $\square$

**The probability density of transfer matrices** The following lemma builds on the arguments going back to the work of Ricci and Stein [33]. In the context of random Schrödinger operators, similar reasoning appears in the work Shubin, Vakilian and Wolff [35]. Recently, a general argument in the setting of motivic morphisms has been developed by Glazer and Hendel [11]; further arguments are discussed in [12]. For completeness, we sketch a proof (restricted to the generality of the current discussion) below.

### Lemma 2.3

Assume Assumption 1.1. There exists $n_0$ such that the following holds.

- For any $\lambda \in {\mathbb {R}}$ the distribution of $\Phi _{n_0}(\lambda )$ is absolutely continuous with bounded density with respect to the Haar measure on ${\text {Sp}}(2W, {\mathbb {R}})$.
- Let $\Phi _n(\lambda ) = U_n(\lambda ) \Sigma _n(\lambda ) V_n(\lambda )^*$ be the singular value decomposition of $\Phi _n(\lambda )$. Then there exists *C* such that for any $n \ge n_0$ the distributions of $V_n(\lambda )$, $U_n(\lambda )$ and $V_n^*(\lambda ) U(\lambda )$ are absolutely continuous with density $\le C$ with respect to the Haar measure on the compact symplectic group ${\text {Sp}}(2W, {\mathbb {R}}) \cap {\text {SO}}(2W, {\mathbb {R}})$.

Moreover, the bounds in (a)–(b) are locally uniform in $\lambda$.

### Remark 2.4

For concreteness, we may assume that the singular value decomposition is constructed so that $\Sigma _n$ is diagonal with strictly decreasing positive entries on the diagonal, and the first non-zero entry of eich column of $U_n$ and $V_n$ is positive.

### Proof

Consider the product map

20 $$\begin{aligned} F_{n} = F_{n, \lambda }: {\mathcal {M}}^n \rightarrow {\text {Sp}}(2W, {\mathbb {R}}), \quad (V(1), \cdots , V(n)) \mapsto \Phi _{n}(\lambda ). \end{aligned}$$

According to [33, Proposition 1.1], for

$$\begin{aligned} n_1 = 2^{\dim {\text {Sp}}(2W, {\mathbb {R}}) - \dim {\mathcal {M}}} = 2^{W(2W+1) - \dim {\mathcal {M}}} \end{aligned}$$

the image $F_{n_1}({\mathcal {M}})$ contains an open set in ${\text {Sp}}(2W, {\mathbb {R}})$ (in the Schrödinger case, the same conclusion holds for $n_1 = \dim {\text {Sp}}(2W, {\mathbb {R}}) \div \dim {\mathcal {M}} = 2W+1$; see [29, Proposition 1.4.35]). Hence $\det [(D F_{n_1})^* (D F_{n_1})]$ is not identically zero; by continuity, the maximum of its absolute value is bounded away from zero locally uniformly in $\lambda$.

The map (20) is real analytic, therefore the probability density of $\Phi _{n_1}(\lambda )$ lies in $L_p$ for some $p>1$ (this can be proved directly as in [12] or deduced from [33, Proposition 2.1] using an appropriate embedding theorem), and, again, both *p* and the bound are locally uniform in $\lambda$. Applying the inequality

$$\begin{aligned} \Vert f_1 * f_2 * \cdots * f_n \Vert _\infty \le \prod _{\alpha =1}^n \Vert f_\alpha \Vert _{1+\frac{1}{n}}, \quad f_\alpha \in L_{1 + \frac{1}{n}}({\text {Sp}}(2W, {\mathbb {R}})) \end{aligned}$$

(which is a simple special case of the Young convolution inequality on ${\text {Sp}}(2W, {\mathbb {R}})$), we obtain that for $n_0= n_1 (\lfloor (1 - 1/p)^{-1} \rfloor + 1)$ the density of $\Phi _{n_0}(\lambda )$ is bounded. This proves the first item, from which the second one follows. $\square$

**A geometric lemma** Denote by *S*(*F*) the unit sphere of an Euclidean vector space *F*. For future reference, we record the following fact (attributed to Archimedes): if *u* is a random vector uniformly distributed on $S({\mathbb {R}}^\ell )$, then the probability density of the random vector $P_F u$, where $P_F: {\mathbb {R}}^\ell \rightarrow F$ be the orthogonal projection onto a fixed *k*-dimensional subspace $F \subset {\mathbb {R}}^\ell$, $1 \le k \le \ell - 1$, is given by

21 $$\begin{aligned} f_{\ell ,k}(v) = C_{\ell ,k} (1 - \Vert v\Vert )_+^{\frac{\ell -k}{2} - 1}.\end{aligned}$$

### Lemma 2.5

Let *U* be a random matrix taking values in ${\text {SO}}(\ell , {\mathbb {R}})$ such that for each $u \in S({\mathbb {R}}^\ell )$ the vector *Uu* is uniformly distributed on $S({\mathbb {R}}^\ell )$. Let $D = {\text {diag}}(e^{a_1}, \cdots , e^{a_\ell })$, where $a_1 \ge a_2 \ge \cdots \ge a_\ell$, and let $F \subset {\mathbb {R}}^\ell$ be a *k*-dimensional subspace. Then for any $a_1 \ge a \ge a_\ell$

$$\begin{aligned} {\mathbb {P}} \left\{ \exists u \in S(F) \, : \, \Vert D U u \Vert \le e^a \right\} \le C_\ell \exp \left\{ - \sum _{j=k}^\ell (a_j - a)_+\right\} .\end{aligned}$$

### Proof

It is sufficient to prove the estimate for the $\ell _\infty$ norm $\Vert \cdot \Vert _\infty$ in place of the Euclidean norm, as this will only affect the value of the numerical constant $C_\ell$. We first observe that for a fixed $u \in S({\mathbb {R}}^\ell )$

22 $$\begin{aligned} {\mathbb {P}} \left\{ \Vert DU u\Vert _\infty \le e^{a} \right\} \le C_\ell \exp (-\sum _{j=1}^\ell (a_j - a)_+). \end{aligned}$$

Indeed, let $j_0$ be such that $a_{j_0} \ge a > a_{j_0+1}$. The random vector $((Uu)_j)_{j=1}^{j_0}$ has bounded density in a neighbourhood of zero (according to (21), for $j_0 \le \ell - 2$ the density is uniformly bounded, whereas for $j_0 = \ell - 1$ it explodes only on the boundary of the unit ball). Therefore

$$\begin{aligned}&{\mathbb {P}} \left\{ \Vert DU u\Vert _\infty \le e^{a} \right\} = {\mathbb {P}} \left\{ \forall 1 \le j \le j_0 \,\, |(DU u)_j| \le e^{a} \right\} \\&\quad \le C_\ell \prod _{j=1}^{j_0} e^{a -a_j} = C_\ell \exp (-\sum _{j=1}^\ell (a_j - a)_+),\end{aligned}$$

thus concluding the proof of (22).

Second, we note that if $\Vert Dv\Vert _\infty \le e^a$, then $\Vert Dv'\Vert _\infty \le 2e^a$ for all

$$\begin{aligned} v' \in Q_v = \{ v' \in S({\mathbb {R}}^\ell ) \, : \, |v_j' - v_j| \le \exp (- (a_j - a)_+)\}. \end{aligned}$$

For any *k*-dimensional subspace $F_1 \subset {\mathbb {R}}^\ell$ and $v \in S(F_1)$, the $k-1$ dimensional measure of the intersection of $Q_v$ with $S(F_1)$ admits the lower bound

$$\begin{aligned} \sigma _{k-1} (S(F_1) \cap Q_v) \ge c_\ell \exp (- \sum _{j=1}^{k-1} (a_j - a)_+),\end{aligned}$$

whence by the Chebyshev inequality, the Fubini theorem and (22)

$$\begin{aligned}\begin{aligned}&{\mathbb {P}} \left\{ \exists v \in S(UF) \, : \, \Vert Dv\Vert _\infty \le e^a \right\} \\&\quad \le {\mathbb {P}} \left\{ \sigma _{k-1} \left\{ v' \in S(UF) \, : \, \Vert Dv'\Vert _\infty \le 2e^a\right\} \ge c_\ell \exp (-\sum _{j=1}^{k-1} (a_j - a)_+)\right\} \\&\quad \le C_\ell ' \exp (\sum _{j=1}^{k-1}(a_j - a)_+) {\mathbb {E}} \sigma _{k-1} \left\{ v' \in S(UF) \, : \, \Vert Dv'\Vert _\infty \le 2e^a\right\} \\&\quad \le C_\ell '' \exp (\sum _{j=1}^{k-1}(a_j - a)_+) \, \exp (-\sum _{j=1}^\ell (a_j - a)_+) =C_\ell '' \exp \left\{ - \sum _{j=k}^\ell (a_j - a)_+\right\} .\end{aligned} \end{aligned}$$

$$\square $$

## Proof of Theorem 1

For the whole proof, we fix $\lambda \in \sigma (H)$ and $\gamma > \gamma _{*,1}(\lambda )$. Choose an auxiliary small parameter $\epsilon > 0$; eventually, we shall substitute $\epsilon = \frac{1}{100 W} \min (\gamma - \gamma _{*,1}(\lambda ), 1)$.

Denote

23 $$\begin{aligned} \Omega _{n,\epsilon }(\lambda ) = \bigcap _{1 \le j \le W} \bigcap _{1 \le m_1 \le m_2 \le n} \Omega _{n,\epsilon }^{m_1,m_2,j}(\lambda ), \end{aligned}$$

where

$$\begin{aligned} \Omega _{n,\epsilon }^{m_1,m_2,j}(\lambda )&= \left\{ \forall \lambda ' \in (\lambda - r_\epsilon (\lambda ), \lambda + r_\epsilon (\lambda )) \,\,\, \sum _{i=1}^j \log s_i(\Phi _{m_2,m_1}(\lambda '))\right. \\&\left. \le (m_2 - m_1) \sum _{i=1}^j \gamma _i(\lambda ) + 2\epsilon j n \right\} ~ . \end{aligned}$$

From Lemma 2.2 (and using that $\Phi _{m_2, m_1}$ has the same distribution as $\Phi _{m_2-m_1}$) we obtain the following maximal inequality:

24 $$\begin{aligned} {\mathbb {P}}(\Omega _{n,\epsilon }(\lambda ))\ge 1 - C n^3 \exp (-c \epsilon ^2 n). \end{aligned}$$

Let

25 $$\begin{aligned} F_0 = \left\{ \left( {\begin{array}{c}v_1\\ 0\end{array}}\right) \, : \, v_1 \in {\mathbb {R}}^W \right\} \subset {\mathbb {R}}^{2W}\end{aligned}$$

be the space of initial conditions. Denote:

26 $$\begin{aligned} {\text {Fast}}_{n,\epsilon }(\gamma , \lambda ) = \left\{ \lambda ' \in (\lambda - r_\epsilon (\lambda ), \lambda + r_\epsilon (\lambda )) \,\, : \,\, \exists v \in S(F_0), \,\, \Vert \Phi _n(\lambda ') v\Vert \le e^{-n \gamma }\right\} ,\nonumber \\ \end{aligned}$$

so that for any ${\tilde{\gamma }} > \gamma$

$$\begin{aligned} {\text {Fast}}^+\big ({\tilde{\gamma }}, (\lambda - r_\epsilon (\lambda ), \lambda + r_\epsilon (\lambda ))\big ) \subset \limsup _{n \rightarrow \infty } {\text {Fast}}_{n,\epsilon }(\gamma , \lambda ).\nonumber \\ \end{aligned}$$

We shall prove that for sufficiently small $\epsilon$

27 $$\begin{aligned} {\mathbb {P}} \left\{ {\text {Fast}}_{n,\epsilon }(\gamma , \lambda ) \ne \varnothing \right\} \le C e^{-cn}~; \end{aligned}$$

by the Borel–Cantelli lemma, this estimate will imply that almost surely

$$\begin{aligned} {\text {Fast}}^+\big ({\tilde{\gamma }}, (\lambda - r_\epsilon (\lambda ), \lambda + r_\epsilon (\lambda ))\big ) = \varnothing , \quad {\tilde{\gamma }} > \gamma ,\end{aligned}$$

and thus $$\gamma _*^+ \le \gamma $$.

The proof of (27) rests on two claims, a propagation estimate and a single-energy bound. Set $\eta = n^{-1} e^{-n(\gamma + \gamma _1(\lambda ) + 4 \epsilon )}$.

### Claim 3.1

On the event $\Omega _{n,\epsilon }(\lambda )$,

28 $$\begin{aligned} \left. \begin{aligned} \lambda ', \lambda '' \in (\lambda - r_\epsilon (\lambda ), \lambda + r_\epsilon (\lambda ))&\\ \lambda ' \in {\text {Fast}}_{n,\epsilon }(\gamma , \lambda )&\\ |\lambda '' - \lambda '| \le \eta&\end{aligned} \right\} \Longrightarrow \lambda '' \in {\text {Fast}}_{n,\epsilon }(\gamma - \frac{\log 2}{n}, \lambda ). \end{aligned}$$

### Proof

On $\Omega _{n,\epsilon }(\lambda )$, we have

29 $$\begin{aligned} \lambda ', \lambda '' \in (\lambda - r_\epsilon (\lambda ), \lambda + r_\epsilon (\lambda )) \Longrightarrow \Vert \Phi _n(\lambda ') - \Phi _n(\lambda '') \Vert \le n e^{n (\gamma _1(\lambda ) + 4\epsilon )} |\lambda ' - \lambda ''|, \nonumber \\ \end{aligned}$$

hence for $|\lambda ' - \lambda ''| \le \eta$ we have

$$\begin{aligned} \Vert \Phi _n(\lambda ') - \Phi _n(\lambda '')\Vert \le e^{-n\gamma }. \end{aligned}$$

If $\lambda ' \in {\text {Fast}}_{n,\epsilon }(\gamma , \lambda )$, then there exists $v \in S(F_0)$ such that $\Vert \Phi _n(\lambda ') v\Vert \le e^{-n\gamma }$, and then

30 $$\begin{aligned} \Vert \Phi _n(\lambda '') v\Vert \le e^{-n\gamma } + \Vert \Phi _n(\lambda '')-\Phi _n(\lambda ')\Vert \le e^{-n\gamma } + e^{-n\gamma } = 2e^{-n\gamma }, \end{aligned}$$

i.e. $$\lambda '' \in {\text {Fast}}_{n,\epsilon }(\gamma - \frac{\log 2}{n}, \lambda )$$, as asserted.$$\square $$

### Claim 3.2

For any $\gamma >0$, $n \ge n_0$, and $\lambda '' \in (\lambda - r_\epsilon (\lambda ), \lambda +r_\epsilon (\lambda ))$

31 $$\begin{aligned} \begin{aligned}&{\mathbb {P}} \left\{ \lambda '' \in {\text {Fast}}_{n,\epsilon }(\gamma , \lambda ), \, \omega \in \Omega _{n,\epsilon }(\lambda ) \right\} \\&\qquad \le C \exp \left\{ -n \left[ 2 \gamma + \big ( (W-1)\gamma - \sum _{j=1}^{W-1} \gamma _{j}(\lambda ) \big )_+ - 2W\epsilon \right] \right\} . \end{aligned}\end{aligned}$$

### Proof

Let $n_0$ be as in Lemma 2.3, and let $M \in {\text {Sp}}(2W, {\mathbb {R}})$ be a random matrix uniformly distributed according to the restriction of the Haar measure to a sufficiently large ball in operator norm. Denote ${\widetilde{\Phi }}_n(\lambda ) = \Phi _{n, n_0}(\lambda )M$. According to Lemma 2.3, it suffices to show that32Introduce the singular value decompositon

$$\begin{aligned} \Phi _{n, n_0}(\lambda '') = U_{n, n_0}(\lambda '') \Sigma _{n, n_0}(\lambda '') V_{n, n_0}(\lambda '')^*, \quad M =U \Sigma V^*,\end{aligned}$$

so that

$$\begin{aligned}{\widetilde{\Phi }}_n(\lambda '') = U_{n, n_0}(\lambda '') \Sigma _{n, n_0}(\lambda '') \left[ V_{n, n_0}(\lambda '')^*U \right] \Sigma V^*,\end{aligned}$$

and let $F_1 = \Sigma V^* F_0$. If $\Vert {\widetilde{\Phi }}_n(\lambda '') v_0 \Vert \le e^{-n\gamma }$ for some $v_0 \in S(F_0)$, then

33 $$\begin{aligned} \Vert \Sigma _{n, n_0}(\lambda '') \left[ V_{n, n_0}(\lambda '')^*U\right] v_1 \Vert \le e^{-n\gamma + C_1} \end{aligned}$$

for $v_1 = \Sigma V^* v_0 / \Vert \Sigma V^* v_0 \Vert \in S(F_1)$. Note that $\left[ V_{n, n_0}(\lambda '')^*U\right]$ is distributed uniformly on the compact symplectic group, and therefore its action on any fixed vector on the sphere is distributed uniformly on the sphere. On the event $\Omega _{n,\epsilon }(\lambda )$, the numbers $a_j = \frac{1}{n} \log s_j(\Phi _{n_0, n}(\lambda ))$ satisfy

$$\begin{aligned} a_{2W+1-j}&= - a_j, \quad \sum _{i=1}^j a_i \le (1 - n_0/n) \sum _{i=1}^j \gamma _i(\lambda ) + 2\epsilon j \\&\le \sum _{i=1}^j \gamma _i(\lambda ) + 2\epsilon W \quad (1 \le j \le W). \end{aligned}$$

Therefore

$$\begin{aligned} \begin{aligned} \sum _{j=1}^{W+1} \left( \gamma - a_j\right) _+&\ge 2 \gamma +\sum _{j=1}^{W-1} (\gamma - a_j)_+ \\&\ge 2 \gamma + (W-1) \big (\gamma - \frac{1}{W-1} \sum _{j=1}^{W-1} a_j\big )_+ \ge 2 \gamma \\&\quad + \big ((W-1)\gamma - \sum _{j=1}^{W-1} \gamma _j(\lambda )\big )_+ - 2 \epsilon W,\end{aligned}\end{aligned}$$

whence

$$\begin{aligned} \sum _{j=1}^{W+1} (\gamma - \frac{C_1}{n} - a_j)_+ \ge 2 \gamma + \big ((W-1)\gamma - \sum _{j=1}^{W-1} \gamma _j(\lambda )\big )_+ - 2 \epsilon W - \frac{2C_1W}{n} \end{aligned}$$

According to Lemma 2.5,as claimed in (32). $\square$

Now we combine Claim 3.1 with Claim 3.2 (applied to $\gamma - \log 2 /n$ in place of $\gamma$) and (24), and use the Fubini theorem:

$$\begin{aligned} \begin{aligned}&{\mathbb {P}} \left\{ {\text {Fast}}_{n,\epsilon }(\gamma , \lambda ) \ne \varnothing \right\} \\&\quad \le (1 - {\mathbb {P}} (\Omega _{n,\epsilon }(\lambda )) + 2 C r_\epsilon (\lambda ) \\&\quad \exp \left\{ - n \left[ 2 \gamma + ((W-1)\gamma - \sum _{j=1}^{W-1} \gamma _j(\lambda ))_+ - 2 \epsilon W \right] \right\} \eta ^{-1} \\&\quad \le C n^3 e^{-cn} + C n \\&\quad \exp \left\{ - n \left[ - \gamma _1(\lambda ) + \gamma + ((W-1)\gamma - \sum _{j=1}^{W-1} \gamma _j(\lambda ))_+ - 4 \epsilon W \right] \right\} . \end{aligned}\end{aligned}$$

For $\epsilon = \frac{1}{100 W} \min (\gamma - \gamma _{*,1}, 1)$, this expression tends to zero exponentially with *n*, thus concluding the proof of (27) and of Theorem 1. $\square$

## Proof of Theorem 2

Let $\gamma > \frac{1}{3} (2\gamma _1(\lambda )+\gamma _2(\lambda ))$, and let $\epsilon = \frac{1}{100} \min (\gamma _1(\lambda ) - \gamma _2(\lambda ), \gamma _2(\lambda ), 1)$. We keep the notation $F_0$ (space of initial conditions, (25)), $\Omega _{n,\epsilon }(\lambda )$ (the event on which the products of singular values admit an upper bound, (23)), and ${\text {Fast}}_{n,\epsilon }(\gamma , \lambda )$ (the set of energies $\lambda '$ in the vicinity of $\lambda$ for which there is a fast-decaying solution, (26)) from the previous section. Similarly to the previous section, or goal is to prove (27), i.e. that ${\text {Fast}}_{n,\epsilon }(\gamma , \lambda )$ is empty outside an event of exponentially small probability.

Denote by $u_j(\lambda ')$ ($j=1,2,3,4$) the right singular vectors of $\Phi _n(\lambda ')$ (i.e. the eigenvectors of $\Phi _n(\lambda ')^* \Phi _n(\lambda ')$; the choice of the direction of the vectors will be specified later), and by $P_{F_0}$ – the orthogonal projection onto $F_0$. Let

34 $$\begin{aligned}&\eta = \frac{1}{n} \exp (-n(\gamma - \gamma _2(\lambda ))), \end{aligned}$$

35 $$\begin{aligned}&A^+ = \left\{ \lambda '' \in (\lambda - r_\epsilon (\lambda ), \lambda + r_\epsilon (\lambda )) \, : \, \Vert P_{F_0} u_1(\lambda '') \Vert \le C \exp (-n(2\gamma -\gamma _1-\gamma _2-4\epsilon ))\right\} ,\nonumber \\ \end{aligned}$$

where $C>0$ will be specified shortly. The required estimate (27) follows from (24) and the following two ingredients: a propagation estimate36which replaces Claim 3.1, and the single-energy bound

37 $$\begin{aligned} |\lambda '' - \lambda | < r_\epsilon (\lambda ) \Longrightarrow {\mathbb {P}} \left\{ \lambda '' \in A^+\right\} \le C' e^{-\epsilon n} \eta \nonumber \\ \end{aligned}$$

which replaces Claim 3.2.

To prove (36), we first observe that $\lambda '\in A$ implies that $\frac{1}{n} \log s_1(\Phi _n(\lambda ')) \ge \gamma$, and hence on $\Omega _{n,\epsilon }(\lambda )$

38 $$\begin{aligned} \begin{aligned} \frac{1}{n} \log s_2(\Phi _n(\lambda '))&= \frac{1}{n} \big (\log s_1(\Phi _n(\lambda ')) + \log s_2(\Phi _n(\lambda '))\big )- \frac{1}{n} \log s_1(\Phi _n(\lambda ')) \\&\le \gamma _1(\lambda ) + \gamma _2(\lambda ) - \gamma + 4\epsilon . \end{aligned}\end{aligned}$$

Further, $\lambda ' \in A$ implies that there exists $v \in F_0$ such that for $j=1,2,3$

$$\begin{aligned} |\langle v, u_j(\lambda ') \rangle |\le & {} \frac{\exp (- n \gamma )}{s_j(\Phi _n(\lambda '))} \le \exp (- n \gamma ) s_2(\Phi _n(\lambda '))\\\le & {} \exp (- n (2\gamma - \gamma _1(\lambda ) - \gamma _2(\lambda ) - 4\epsilon ) ).\end{aligned}$$

These inequalities imply that (for the appropriate choice of signs)

$$\begin{aligned} \Vert v - u_4(\lambda ') \Vert \le C_1 \exp (- n (2\gamma - \gamma _1(\lambda ) - \gamma _2(\lambda ) - 4\epsilon ) ).\end{aligned}$$

Now we use the symplectic rotation $$J = \left( \begin{array}{cc} 0 &{} - \mathbbm {1} \\ \mathbbm {1} &{} 0 \end{array} \right) $$. The matrix $\Phi _n(\lambda ')$ is symplectic, hence (up to sign) $J u_4(\lambda ') = u_1(\lambda ')$. Thus

$$\begin{aligned} \Vert J v - u_1(\lambda ') \Vert \le C_1 \exp (- n (2\gamma - \gamma _1(\lambda ) - \gamma _2(\lambda ) - 4\epsilon ) ).\end{aligned}$$

On the other hand, $F_0 \subset {\mathbb {R}}^{2W}$ is a Lagrangian subspace (i.e. $F_0 = (J F_0)^\perp$), hence $J v \perp F_0$. Consequently,

39 $$\begin{aligned} \Vert P_{F_0} u_1(\lambda ') \Vert \le C_1 \exp (- n (2\gamma - \gamma _1(\lambda ) - \gamma _2(\lambda ) - 4\epsilon ) ).\end{aligned}$$

To complete the proof of (36), we need to show that the estimate (39) does not deteriorate too fast as we vary $\lambda '$. If $|\lambda '' - \lambda '| \le \eta$ and $|\lambda '' - \lambda | \le r_\epsilon (\lambda )$, we have (still on $\Omega _{n,\epsilon }(\lambda )$, cf. (29)):

$$\begin{aligned} \Vert \Phi (\lambda '') - \Phi (\lambda ') \Vert \le \eta n \exp (n(\gamma _1(\lambda )+4\epsilon )), \end{aligned}$$

whence by Wedin’s perturbation bound for singular vectors [38]

40 $$\begin{aligned} \Vert u_1(\lambda '') - u_1(\lambda ')\Vert\le & {} C_2 \frac{\eta n e^{n(\gamma _1(\lambda )+4\epsilon )}}{s_1(\Phi _n(\lambda ')) - s_2(\Phi _n(\lambda '))}\nonumber \\\le & {} \frac{2C_2 \eta n e^{n(\gamma _1(\lambda )+4\epsilon )}}{e^{n\gamma }} = 2C_2 \exp (-n(2\gamma - \gamma _1(\lambda ) - \gamma _2(\lambda ) - 4\epsilon )). \nonumber \\ \end{aligned}$$

On the second step we used that $s_1(\Phi _n(\lambda ')) - s_2(\Phi _n(\lambda ')) \ge \frac{1}{2} e^{n \gamma }$. This estimate holds (for sufficiently large *n*) since

$$\begin{aligned} s_1(\Phi _n(\lambda ')) \ge e^{n\gamma }, \quad s_1(\Phi _n(\lambda )) s_2(\Phi _n(\lambda )) \le e^{n(\gamma _1(\lambda ) + \gamma _2(\lambda ) + 4 \epsilon )}, \end{aligned}$$

whereas

$$\begin{aligned}\gamma> \frac{2\gamma _1(\lambda ) + \gamma _2(\lambda )}{3}> \frac{\gamma _1(\lambda ) + \gamma _2(\lambda )}{2}.\end{aligned}$$

From (39) and (40) we obtain that $\lambda '' \in A^+$, provided that we set $C = C_1 + 2C_2$ in (35). This concludes the proof of (36).

To prove (37), we use once again that if *U* is uniformly distributed on the compact symplectic group ${\text {Sp}}(2W, {\mathbb {R}}) \cap {\text {SO}}(2W, {\mathbb {R}})$, then each column of *U* is uniformly distributed on the unit sphere. Thus, according to Lemma 2.3, the probability density of $u_1(\lambda '')$ with respect to the Haar measure on $S({\mathbb {R}}^{2W})$ is bounded uniformly in $n \ge n_0$. Hence by (21)

$$\begin{aligned} \begin{aligned}&{\mathbb {P}} \left\{ \Vert P_{F_0} u_1(\lambda '') \Vert \le C \exp (-n(2\gamma -\gamma _1-\gamma _2-4\epsilon )) \right\} \\&\quad \le C_4 \exp (-2n(2\gamma -\gamma _1-\gamma _2-4\epsilon )) \\&\quad \le C_5 \exp (-\epsilon n) \eta .\end{aligned}\end{aligned}$$

This concludes the proof of (37) and of the theorem. $\square$

## On the Uniform Convergence to the Lyapunov Exponents

A better understanding of the deviations of $\frac{1}{n} \log s_j(\Phi _n(\lambda ))$ from their limiting values $\gamma _j$ would allow us to strengthen the conclusion of Theorems 1 and 2, possibly up to the conjectured $\gamma _*^+ \overset{\text {conj}}{=} \gamma _1$, as we now discuss.

Recall the following result from [13, 14] pertaining to $W=1$: with probability one, the set $\Lambda _{\frac{1}{2}}$, where

$$\begin{aligned} \Lambda _{\tau } = \left\{ \lambda \in {\mathbb {R}}\, : \, \liminf _{n \rightarrow \infty } \frac{1}{n} \log \Vert \Phi _n(\lambda ) \Vert \le \tau \gamma _1(\lambda )\right\} , \quad \tau \in [0,1], \end{aligned}$$

is dense in $\sigma (H)$. Subsequently, it was found that also the (possibly) smaller set $\Lambda _{0}$ is almost surely dense in $\sigma (H)$. Recently, a general framework encompassing and generalising these results was developed by Gorodetski and Kleptsyn [19], who also provided detailed information on the structure of the exceptional sets $\Lambda _\tau$, and showed that

41 $$\begin{aligned} {\mathbb {P}} \left\{ \forall \lambda \in {\mathbb {R}}\, : \, \limsup _{n \rightarrow \infty } \frac{1}{n} \log \Vert \Phi _n(\lambda ) \Vert = \gamma _1(\lambda )\right\} = 1. \end{aligned}$$

We are not aware of a published reference discussing the extension of this problem for $W > 1$. However, it is plausible that the arguments developed in the aforementioned works could yield that

$$\begin{aligned} \Lambda _{0}^{(W)} = \left\{ \lambda \in {\mathbb {R}}\, : \, \liminf _{n \rightarrow \infty } \frac{1}{n} \log s_W(\Phi _n(\lambda )) =0 \right\} \end{aligned}$$

is dense in $\sigma (H)$. It is not clear to us what would be the right counterpart of this statement for $1 \le j \le W-1$. If the higher exponents would exhibit regular behaviour, i.e.

42 $$\begin{aligned}&\text {if it were true that } {\mathbb {P}} \left\{ \forall \lambda \in \sigma (H) \quad \lim _{n \rightarrow \infty } \frac{1}{n} \log s_j(\Phi _n(\lambda )) = \gamma _j(\lambda ) \right\} \nonumber \\&\quad =1, \quad 1 \le j \le W-1, \end{aligned}$$

one could significantly improve the results of the current paper: the argument of Theorem 1 would yield $\gamma _*^+ \le \gamma _{*,2}$, where $\gamma _{*,2}$ is the solution of

$$\begin{aligned} \gamma + \sum _{j=1}^{W-1} \left( \gamma - \gamma _j\right) _+ = \gamma _1, \end{aligned}$$

whereas the argument of Theorem 2 would establish the optimal bound $\gamma _*^+ = \gamma _W$ (for arbitrary *W*, cf. the proof of Theorem 3 below). If (42) is false, it would be helpful to understand

43 $$\begin{aligned} \text {is it true that \,} {\mathbb {P}} \left\{ \forall \lambda \in \sigma (H) \quad \limsup _{n \rightarrow \infty } \frac{1}{n} \log s_j(\Phi _n(\lambda )) \le \gamma _j(\lambda ) \right\} =1, \quad 1 \le j \le W.\nonumber \\ \end{aligned}$$

Following Craig and Simon [7] (cf. Lemma 2.2), note that (43) holds (unconditionally) for $j=1$. Also (according to the same lemma) (42) would imply (43).

In this section, we prove the following extension of (41) to 2*W*-dimensional cocycles. We confine ourselves to the setting of transfer matrices, which is used in the proof of Theorem 3. Denote

$$\begin{aligned} {\text {Dev}}_{n}(\lambda ) = \max _{1 \le j \le W} \left| \frac{1}{n} \log s_j(\Phi _n(\lambda )) - \gamma _j(\lambda )\right| .\end{aligned}$$

### Proposition 5.1

Assume that *V*(*n*) satisfy (1), and that (8) holds for every $\lambda \in [a,b]$. Then for any $\epsilon > 0$ there exist $C> 0$ and $c > 0$ such that

44 $$\begin{aligned} {\mathbb {P}} \left\{ \sup _{\lambda \in [a,b]} \min \left( {\text {Dev}}_n(\lambda ), {\text {Dev}}_{n^2}(\lambda )\right) \ge \epsilon \right\} \le C e^{-cn}. \end{aligned}$$

In particular,

$$\begin{aligned} {\mathbb {P}} \left\{ \sup _{\lambda \in [a,b]} \liminf _{n \rightarrow \infty } {\text {Dev}}_n(\lambda )= 0 \right\} = 1. \end{aligned}$$

### Remark 5.2

Here $n^2$ can be replaced with any function tending to infinity faster than linearly.

### Proof of Proposition 5.1

Fix $\lambda \in {\mathbb {R}}$; let $\epsilon > 0$, and choose $r_\epsilon (\lambda )$ as in (17). It will suffice to show that there exist *C*, *c* such that

45 $$\begin{aligned} {\mathbb {P}} \left\{ \sup _{|\lambda ' - \lambda | < r_\epsilon (\lambda )} \min \left( d_n(\lambda '), d_{n^2}(\lambda ')\right) \ge 10 W \epsilon \right\} \le C e^{-cn}, \end{aligned}$$

where

46 $$\begin{aligned} d_n(\lambda ') = \max _{1 \le j \le n} \left| \frac{1}{n} \log s_j(\Phi _n(\lambda ')) - \gamma _j(\lambda )\right| .\end{aligned}$$

By the existence of a fractional moment (1) and the Chebyshev inequality, one can choose $\kappa > 0$ such that

$$\begin{aligned} {\mathbb {P}} (\Omega ^{(1)}_n) \ge 1 - e^{-n}, \quad \Omega ^{(1)}_n = \left\{ \sup _{|\lambda ' - \lambda | < r_\epsilon (\lambda )} \Vert \Phi _n(\lambda ')\Vert \le e^{\kappa n} \right\} .\end{aligned}$$

On $$\Omega ^{(1)}_n$$,

$$\begin{aligned} \left| \log s_j(\Phi _{n^2}(\lambda ')) - \log s_j(\Phi _{n^2, n}(\lambda ')) \right| \le \kappa n,\end{aligned}$$

therefore for sufficiently large *n*

$$\begin{aligned} d_{n^2}(\lambda ') \le \epsilon + {\tilde{d}}_{n^2}(\lambda '), \quad {\tilde{d}}_{n^2}(\lambda ') = \max _{1 \le j \le n} \left| \frac{1}{n^2-n} \log s_j(\Phi _{n^2,n}(\lambda ')) - \gamma _j(\lambda )\right| . \end{aligned}$$

Here ${\tilde{d}}_{n^2}(\cdot )$ is independent of $d_n(\cdot )$. Also recall from Lemma 2.2 that ${\mathbb {P}}(\Omega _n^{(2)}) \ge 1 - C e^{-cn}$, where

$$\begin{aligned}&\Omega _n^{(2)} = \left\{ \forall \lambda ' \in (\lambda - r_\epsilon (\lambda ), \lambda + r_\epsilon (\lambda )), \, 1 \le j \le n\,\, : \,\,\right. \\&\quad \left. \frac{1}{n} \sum _{i=1}^j \log s_i(\Phi _n(\lambda ')) \le \sum _{i=1}^j \gamma _i(\lambda ) + 2j \epsilon \right\} .\end{aligned}$$

Now, Lemma 2.1 implies that for each $\lambda '\in (\lambda - r_\epsilon (\lambda ), \lambda + r_\epsilon (\lambda ))$

47 $$\begin{aligned} {\mathbb {P}} \left\{ |\frac{1}{n} \log |||\Phi _n(\lambda ')||| - \gamma _1(\lambda ) | \ge 2\epsilon \right\} \le C \exp (-c n), \end{aligned}$$

where $|||X||| = \max _{\alpha ,\beta } |X_{\alpha ,\beta }|$ (the $\ell _1$ to $\ell _\infty$ norm). Note that each matrix entry of $\Phi _n(\lambda ')$ is a polynomial in $\lambda '$ of degree *n*, therefore the set $A_n^{(1)}$ of $\lambda ' \in (\lambda - r_\epsilon (\lambda ),\lambda + r_\epsilon (\lambda ))$ for which $|\frac{1}{n} \log |||\Phi _n(\lambda ')||| - \gamma _1(\lambda ) | \ge 2\epsilon$ is a union of at most $W^2 n$ intervals. Applying the same argument to the wedge powers $\Phi _n(\lambda )^{\wedge j}$, we construct the sets $A_n^{(2)}, \cdots , A_n^{(W)}$ such that $A_n^{(j)}$ is a union of at most $C_j(W) n$ intervals (where $C_j(W)$ may depend only on *j* and *W*),

48 $$\begin{aligned} {\mathbb {P}} \left\{ \lambda ' \in A_n^{(j)}\right\} \le C e^{-cn}, \end{aligned}$$

and

$$\begin{aligned} \lambda ' \in (\lambda - r_\epsilon (\lambda ), \lambda + r_\epsilon (\lambda )) \setminus A_n^{(j)} \Longrightarrow \frac{1}{n} \sum _{i=1}^j \log s_i(\Phi _n(\lambda ')) \ge \sum _{i=1}^j \gamma _i(\lambda ) - 2j \epsilon .\end{aligned}$$

We also construct similar sets $A_{n^2,n}^{(j)}$ corresponding to $\Phi _{n^2,n}(\lambda ')$, and let

$$\begin{aligned} A_n = \bigcup _{j=1}^W A_n^{(j)}, \quad A_{n^2,n} = \bigcup _{j=1}^W A_{n^2,n}^{(j)}. \end{aligned}$$

The set $A_n$ is a union of $\le C(W) n$ intervals, whereas $A_{n^2,n}$ is a union of $\le C(W) n^2$ intervals. If these two sets intersect, than either one of the edges of the intervals comprising $A_n$ lies in $A_{n^2,n}$, or vice versa. Invoking (48), we see that

$$\begin{aligned}{\mathbb {P}} (\Omega _n^{(3)}) \ge 1 - C e^{-cn}, \quad \text {where} \quad \Omega _n^{(3)} = \left\{ A_n \cap A_{n^2, n} = \varnothing \right\} . \end{aligned}$$

Observe that on $\Omega _n^{(1)} \cap \Omega _n^{(2)} \cap \Omega _n^{(3)}$, for each $\lambda '$, either $\frac{1}{n} \log s_j(\Phi _n(\lambda '))$ is close to $\gamma _j(\lambda )$ for all *j*, or this holds true for $\frac{1}{n^2} \log s_1(\Phi _{n^2}(\lambda '))$. This concludes the proof of the proposition. $\square$

## Proof of Theorem 3

We keep the notation from the previous sections. Let $\gamma > \gamma _W(\lambda )$, and let $\epsilon = \frac{1}{100 W^2} (\gamma - \gamma _W(\lambda ))$. It suffices to show that

49 $$\begin{aligned} {\mathbb {P}} \left\{ {\text {Fast}}_{n,\epsilon }(\gamma , \lambda ) \cap {\text {Fast}}_{n^2,\epsilon }(\gamma , \lambda ) \ne \varnothing \right\} \le C e^{-cn}. \end{aligned}$$

To keep the notation consistent with the previous sections, it will be convenient to rely on the estimate (45) rather than on the conclusion of Proposition 5.1. Denote

$$\begin{aligned} {\text {Reg}}_{n,\epsilon }(\lambda ) = \left\{ \lambda ' \in (\lambda - r_\epsilon (\lambda ),\lambda + r_\epsilon (\lambda )) \, : \, d_n(\lambda ') < 10W\epsilon \right\} \end{aligned}$$

where $d_n$ are as in (46). From (45),

$$\begin{aligned} {\mathbb {P}}\left\{ {\text {Reg}}_{n,\epsilon }(\lambda ) \cup {\text {Reg}}_{n^2,\epsilon }(\lambda ) = (\lambda - r_\epsilon (\lambda ),\lambda + r_\epsilon (\lambda ))\right\} \ge 1 - C e^{-cn}. \end{aligned}$$

Therefore (49) and the theorem are implied by (24) and the following estimate:

50 $$\begin{aligned} {\mathbb {P}} \left\{ {\text {Fast}}_{n,\epsilon }(\gamma , \lambda ) \cap {\text {Reg}}_{n,\epsilon }(\lambda ) \ne \varnothing ; \omega \in \Omega _{n,\epsilon }(\lambda ) \right\} \le C e^{-cn}. \end{aligned}$$

The proof of (50) is similar to the argument in Sect. 4. Denote

$$\begin{aligned} A = {\text {Fast}}_{n,\epsilon }(\gamma , \lambda ) \cap {\text {Reg}}_{n,\epsilon }(\lambda ), \quad \eta = \frac{1}{n} \exp (- (\gamma - \gamma _W(\lambda ) + 20W^2 \epsilon )n), \end{aligned}$$

and let $A^+$ be the set of $\lambda '' \in (\lambda - r_\epsilon (\lambda ), \lambda + r_\epsilon (\lambda ))$ for which there exists

51 $$\begin{aligned} w \in S({\text {span}}(u_1(\lambda ''), \cdots , u_{W-1}(\lambda ''))), \,\, \Vert P_{F_0} w \Vert \le C \exp (-n (\gamma - \gamma _W(\lambda ) - 10 W \epsilon )),\nonumber \\ \end{aligned}$$

where $C>0$ will be specified later. We claim that on $\Omega _{n,\epsilon }(\lambda )$ we have the propagation estimate

52 $$\begin{aligned} \lambda ' \in A, \,\, |\lambda '' - \lambda '|< \eta ,\,\,|\lambda '' - \lambda | < r_\epsilon (\lambda ) \Longrightarrow \lambda '' \in A^+,\end{aligned}$$

and that for each $\lambda '' \in (\lambda - r_\epsilon (\lambda ), \lambda + r_\epsilon (\lambda ))$

53 $$\begin{aligned} {\mathbb {P}} \left\{ \lambda '' \in A^+ \right\} \le C' \exp (-2n (\gamma - \gamma _W(\lambda ) - 10 W \epsilon )) \end{aligned}$$

These two claims imply (50) and thus conclude the proof of the theorem.

To prove (52), we observe that if $\lambda ' \in A$, there exists $v \in S(F_0)$ such that for all $1 \le j \le W+1$

$$\begin{aligned} | \langle v, u_j(\lambda ') \rangle | \le \frac{\exp (-n \gamma )}{s_j(\Phi _n(\lambda '))} \le \exp (-n(\gamma - \gamma _W(\lambda ) - 10 W \epsilon )), \end{aligned}$$

where $u_j(\lambda ')$ is the *j*-th right singular vector of $\Phi _n(\lambda ')$, and thus there exists $\theta \in S({\mathbb {R}}^{W-1})$ such that

$$\begin{aligned} \Vert v - \sum _{j=1}^{W-1} \theta _j u_{2W+1-j}(\lambda ')\Vert \le C_1 \exp (-n(\gamma - \gamma _W(\lambda ) - 10 W \epsilon )).\end{aligned}$$

Now let $w = Jv$, where *J* is the symplectic rotation. Then $w \perp F_0$, and

54 $$\begin{aligned} \Vert w - \sum _{j=1}^{W-1} \theta _j u_{j}(\lambda ')\Vert \le C_1 \exp (-n(\gamma - \gamma _W(\lambda ) - 10 W \epsilon )). \end{aligned}$$

Applying Wedin’s bound to the *j*-th wedge power of $\Phi _n(\lambda ')$, we have:

$$\begin{aligned}\begin{aligned}&\Vert u_1(\lambda '') \wedge u_2(\lambda '') \wedge \cdots \wedge u_j(\lambda '') - u_1(\lambda ') \wedge u_2(\lambda ') \wedge \cdots \wedge u_j(\lambda ') \Vert \\&\quad \le \frac{C_2 \eta n e^{(\gamma _1(\lambda ) + \cdots + \gamma _j(\lambda ) + 4 W \epsilon )n}}{e^{(\gamma _1(\lambda ) + \cdots + \gamma _j(\lambda ) -10W^2 \epsilon )n}} \le C_2 \eta n e^{12 W^2 \epsilon n} \le C_2 e^{- n(\gamma - \gamma _W(\lambda ) - 10 W \epsilon )},\end{aligned}\end{aligned}$$

and consequently

$$\begin{aligned} \Vert u_j(\lambda '') - u_j(\lambda ')\Vert \le C_3 e^{- n(\gamma - \gamma _W(\lambda ) - 10 W \epsilon )}.\end{aligned}$$

This and (54) implies

55 $$\begin{aligned} \Vert w - \sum _{j=1}^{W-1} \theta _j u_{j}(\lambda '')\Vert \le C_4 \exp (-n(\gamma - \gamma _W(\lambda ) - 10 W \epsilon )), \end{aligned}$$

i.e. $\lambda '' \in A^+$ (if *C* in (51) is chosen appropriately), as claimed in (52).

Now we prove (53). If

56 $$\begin{aligned} \Vert P_{F_0} \sum _{j=1}^{W-1} \theta _j u_{j}(\lambda '') \Vert \le C \exp (-n(\gamma - \gamma _W(\lambda ) - 10 W \epsilon )) \end{aligned}$$

for a certain $\theta \in S({\mathbb {R}}^{W-1})$, then

$$\begin{aligned}\Vert P_{F_0} \sum _{j=1}^{W-1} \theta _j' u_{j}(\lambda '') \Vert \le 2C \exp (-n(\gamma - \gamma _W(\lambda ) - 10 W \epsilon )) \end{aligned}$$

for all $\theta '$ in a neighbourhood of $\theta$ on $S({\mathbb {R}}^{W-1})$; the $(W-2)$-dimensional volume of this neighbourhood is bounded from below by

$$\begin{aligned} c \exp (-(W-2) n(\gamma - \gamma _W(\lambda ) - 10 W \epsilon )). \end{aligned}$$

On the other hand, (21) implies that for each $\theta \ \in S({\mathbb {R}}^{W-1})$

$$\begin{aligned}&{\mathbb {P}} \left\{ \Vert P_{F_0} \sum _{j=1}^{W-1} \theta '_j u_{j}(\lambda '') \Vert \le 2C \exp (-n(\gamma - \gamma _W(\lambda ) - 10 W \epsilon )) \right\} \\&\quad \le C' \exp (-n W(\gamma - \gamma _W(\lambda ) - 10 W \epsilon )). \end{aligned}$$

Therefore the probability that there exists $\theta$ satisfying (56) is at most

$$\begin{aligned} \frac{C'}{c} \exp (-2n (\gamma - \gamma _W(\lambda ) - 10 W \epsilon )),\end{aligned}$$

as claimed. This concludes the proof of (53) and of the theorem. $\square$
